# Reply to Comments on “Increased risk of second cancers at sites associated with HPV after a prior HPV-associated malignancy, a systematic review and meta-analysis”

**DOI:** 10.1038/s41416-019-0440-7

**Published:** 2019-03-26

**Authors:** Duncan C. Gilbert, Claire L. Vale

**Affiliations:** 1 MRC Clinical Trials Unit at UCL, Institute of Clinical Trials and Methodology, 90 High Holborn, London, UK; 2 Sussex Cancer Centre, Royal Sussex County Hospital, Eastern Road, Brighton, UK

**Keywords:** Risk factors, Cancer epidemiology

We thank Péré et al. for their comments and for sharing the data from their CoMPap (Consultation Multidiscipliniare Papillomavirus) programme. The high rate of prior HPV-associated cancers described is in keeping with data from other institutional cohorts (included in the Supplementary data as Table S1^1^). We agree that further study of the cohort of patients who have developed an HPV-associated cancer will define the optimal surveillance strategy and provide further insights into the biology of HPV-associated transformation.

We also thank Jayaraj and Kumarasamy for highlighting what we agree is a key limitation of this study, which is the very small sample size of some of the included studies. Indeed, this precluded a meta-analysis approach for the primary penile cancer. However, we felt that including all studies, even when sample sizes were small, was important in presenting the totality of the data (that included cohorts of relatively rare cancers). We feel we have been explicit throughout regarding all of the limitations of our study,

With respect to the individual weights of the studies, we acknowledge that we did not report individual study weights (as a percentage of the total) on our forest plots. However, we felt both the sizes of the squares representing the individual estimates for each study and the width of the 95% confidence intervals were sufficient to alert readers to the contribution of each individual study to the meta-analysis. Furthermore, we carried out a random effects meta-analysis, which takes into account both the relative size (or weight) of each study in the meta-analysis and the associated heterogeneity. In relation to heterogeneity we agree that there are some limitations with the *I*^2^ statistic, and although we had not previously considered using the Tau-squared statistic we agree it may have been appropriate here.

Jayaraj and Kumarassamy have also queried why we did not evaluate publication bias in the studies included within the meta-analysis; presumably they would have expected to see a funnel plot reported. However, given the extent of heterogeneity observed in our results, we did not feel that such a plot would have been appropriate or helpful, as both heterogeneity and publication bias can cause asymmetry.^2^ Furthermore, as discussed, to avoid or limit the impact of ascertainment bias in our results, we excluded institutional cohorts and studies that did not report SIRs with 95% CI. Although we did not feel any formal investigation of publication bias would have been informative, we did aim to be comprehensive and inclusive in our literature searches and have included tables and description of all eligible studies identified, but not included in the meta-analyses, for completeness.

Finally, we acknowledge that the figure legends correctly refer to standardised incidence ratios; however, in the figures, the axis has been incorrectly labelled as hazard ratio. We are grateful for giving us the opportunity to clarify this.
